# Integrating care for older people with complex needs: key insights and lessons from a seven-country cross-case analysis

**DOI:** 10.5334/ijic.2249

**Published:** 2015-09-23

**Authors:** Walter P. Wodchis, Anna Dixon, Geoff M. Anderson, Nick Goodwin

**Affiliations:** Institute for Health Policy Management and Evaluation, University of Toronto; Toronto Rehabilitation Institute; Institute for Clinical Evaluative Sciences, Toronto, Canada; Centre for Ageing Better, London, UK; Institute for Health Policy Management and Evaluation, University of Toronto; Women's College Research Institute; Institute for Clinical Evaluative Sciences, Toronto, Canada; International Foundation for Integrated Care, London, UK

**Keywords:** integrated care, international comparisons, community and social care, case study, cross-case synthesis

## Abstract

**Background:** To address the challenges of caring for a growing number of older people with a mix of both health problems and functional impairment, programmes in different countries have different approaches to integrating health and social service supports.

**Objective:** The goal of this analysis is to identify important lessons for policy makers and service providers to enable better design, implementation and spread of successful integrated care models.

**Methods:** This paper provides a structured cross-case synthesis of seven integrated care programmes in Australia, Canada, the Netherlands, New Zealand, Sweden, the UK and the USA.

**Key findings:** All seven programmes involved bottom-up innovation driven by local needs and included: (1) a single point of entry, (2) holistic care assessments, (3) comprehensive care planning, (4) care co-ordination and (5) a well-connected provider network. The process of achieving successful integration involves collaboration and, although the specific types of collaboration varied considerably across the seven case studies, all involved a care coordinator or case manager. Most programmes were not systematically evaluated but the two with formal external evaluations showed benefit and have been expanded.

**Conclusions:** Case managers or care coordinators who support patient-centred collaborative care are key to successful integration in all our cases as are policies that provide funds and support for local initiatives that allow for bottom-up innovation. However, more robust and systematic evaluation of these initiatives is needed to clarify the ‘business case’ for integrated health and social care and to ensure successful generalization of local successes.

## Introduction

Across Organization for Economic Cooperation and Development countries life expectancy and survival after the incidence of medical conditions such as cancers or cardiovascular disease continue to increase. In part due to this success, more and more people are living longer and longer with multiple chronic conditions and concomitant functional impairment.

Studies show that about half the US population over 75 has three or more chronic conditions and individuals 85 years and older are six times more likely to have multiple functional impairments than individuals aged 65- to 69-year-olds [1]. Also, the number of older people who are living alone is increasing at the same time the availability of informal care by spouses or family members is declining [2]. These trends create growing demand for health care services to treat multiple chronic medical conditions as well as services to help individuals cope with activities such as dressing, bathing, shopping or food preparation. The latter, commonly referred to as social care services, are often provided by family members or informal care givers but can be provided by formal service providers either as home care services or as part of residential long-term care.

Such formal social care services are often organized and funded separately from health care or medical services, and this can result in fragmented care for people who need both types of services. A common response is to develop integrated health and social care for older people with complex needs. Integrated care can mean different things in different settings, but a common feature is that it seeks to improve the quality of care for individual patients, service users and caregivers by ensuring that services are well coordinated around their needs.

This paper synthesizes evidence from seven case studies of integrated health and social care programmes for older people with complex needs in different countries. The case studies were supported by a project grant from The Commonwealth Fund. The seven countries represented the broad spectrum of performance indicated by the international surveys of patients with complex care needs conducted by The Commonwealth Fund [3]. The purpose of this paper is to identify important lessons from these programmes for policy makers and service providers to enable better design and implementation of integrated care.

### Our approach

Through key contacts in each country we identified integrated care programmes that met the following criteria:

- Population focus on older people with complex needs;
- Process focus on integrating health and social care;
- Community-based models of care;
- Outcome focus on user experience, functional ability, quality or costs (e.g. reduced/prevented use of hospital/acute/institutional settings); and
- Established models of care (not pilots) covering a population/geography.

While we were keen to identify programmes with demonstrated success in achieving at least some of the outcomes of interest, it was not always possible to verify this so we relied to some extent on programmes’ reputation among experts in each country. The organizations running the programmes were approached to ensure their cooperation.

Case studies were written for each programme based on document review and key informant interviews with programme leaders, providers and agencies. Case study authors were identified who had a track record of research in integrated care and were familiar with the programme but independent of the organization delivering care. Many authors had been involved in formal evaluation of the programmes and were able to draw on data even when this is not in the public domain. A template was developed by the research team and authors were asked to complete the following information:

1. Intended aims and objectives;
2. The client population, eligibility, engagement, assessment and care planning processes;
3. Organizational structure and governance for the programme;
4. Integrating activities of the programme; participating providers and agencies;
5. Use of technologies and electronic health records;
6. Programme funding;
7. Evidence of impact, sustainability and spread; and
8. Transferrable lessons for practice and policy.

Case study reports were produced for each of the programmes and published in this issue of the *International Journal of Integrated Care*. This paper provides a cross-case synthesis of the individual programmes based on those case study reports, with the aim of identifying key insights and lessons. There were commonalities in broad aims and challenges across cases but also substantive differences in organizational, functional, professional and service integration as well as population focus. These differences are useful in understanding the varied forms that integration can take. Contrasting the cases provides important lessons for service providers and policy-makers seeking to implement integrated care initiatives.

### Selected case studies

Seven programmes were selected for case studies (summarized in Appendix A):

1. HealthOne Mount Druitt, Sydney, Australia;
2. The Te Whiringa Ora programme in Eastern Bay of Plenty, New Zealand;
3. Geriant, in Noord-Holland province, the Netherlands;
4. Torbay and Southern Devon Health and Care Trust (Torbay), the UK;
5. The Norrtalje Model, Sweden;
6. The Massachusetts General Care Management Program, Boston, USA; and
7. The Program of Research to Integrate the Services for the Maintenance of Autonomy Quebec, Canada.

#### Programme aims and objectives

As Table 1 shows, the programmes have implemented different approaches to support older people with complex health and social care needs. While the programmes’ aims and objectives have similarities, there are important differences among them. Some were primarily designed to improve user experience and independence through greater continuity of home-based care by various professionals (e.g. Program of Research to Integrate the Services for the Maintenance of Autonomy and Norrtalje). Others had a more explicit focus on reducing utilization rates in hospital and in order to reduce costs (e.g. Massachusetts General Care Management Program). Common to all, however, is the recognition that the coordination of care by different professionals should result in better and more cost-effective care outcomes.

#### Client population: eligibility, engagement, assessment and care planning

Each programme has a somewhat distinctive target population. For example, some programmes have defined a very specific older adult client group linked to a clear process for identification and enrolment (e.g., dementia care in Geriant or high-cost service users in Massachusetts General Care Management Program). Other programmes have undertaken a broader and more holistic approach to care by focusing on population-health management for defined communities (e.g., the integrated delivery systems in Program of Research to Integrate the Services for the Maintenance of Autonomy and Norrtalje, or the community-focused approaches in Te Whiringa Ora and Torbay).

Table 2 highlights the approach to assessment and care planning and the engagement of clients and caregivers. All approaches sought to some extent to promote engagement of service users *and* their informal caregivers or family members. Among all programmes, Te Whiringa Ora places the most emphasis on engaging service users and family members as the key to achieving its programme's goals, which are defined by the client (rather than professionals). In Geriant, care managers, clients and informal caregivers jointly make a plan for care treatment each year. HealthOne stresses that patients and caregivers should be active participants in care planning and management and also emphasizes that patients and family participate in case conferences if appropriate and to the extent that they want. In Canada's Program of Research to Integrate the Services for the Maintenance of Autonomy programme clients and family have input in care plans (similar to Geriant), though the emphasis has been to shift from a client focus to population-based care management that provides different levels of support to patient groupings with different levels of need. In some regions, Program of Research to Integrate the Services for the Maintenance of Autonomy patients may also choose a direct payment option where they are given funds to purchase their own care services, an option mostly applied in retirement home settings where in-house services are already available. Torbay and Sweden do not emphasize patient engagement in care planning, but Torbay patients hold yellow folders containing their care plans that they can share with any professional involved in their care. The Mass General programme offers patient-centred care management but patients are not explicitly engaged in the development of the care plans.

#### Organizational structure and governance

We examined many different perspectives on integration across models including: *types* of integration (e.g. organizational, professional); *breadth* of integration (e.g. vertical, horizontal); *level* of integration (including macro- (system), meso- (organizational, professional) and micro-level (service and personal) integration; *degree* of integration (i.e. from linkage to full integration); and *processes* of integration (i.e., cultural and social as well as structural and systemic) [4, 5] (Tables 3 and 4).

We found very different types of integration across the programmes, ranging from ‘fully-integrated’ health and social care providers (e.g. Geriant, Norrtlaje and Torbay) to approaches that have instead sought to build alliances between professionals and providers to co-ordinate care, often based on contractual relationships between otherwise separate partners (e.g. Program of Research to Integrate the Services for the Maintenance of Autonomy, HealthOne, Te Whiringa Ora). In Noortalje (Sweden) a new organization was created to merge the purchasing and provision of health and social care which are otherwise split between municipalities and county councils. It appears that the focus on organizational integration took much time and energy and that changes to services have been slower to develop.

Moreover, while five of the seven programmes seek to integrate both ‘horizontally’ (community-based care coordination) and ‘vertically’ (transitions from hospital to home), two focus largely on the former - mostly through the creation of multidisciplinary teams and enhanced access to community-based services (HealthOne, Te Whiringa Ora). Some programmes combine different types of integration. For example, while Geriant is fully integrated horizontally (i.e., a single organization spans health and social care), it coordinates care vertically (i.e., with hospitals and care homes), and in HealthOne the general practice liaison nurses are involved in clients’ hospital discharge case conferences.

#### Information and communication technologies

Surprisingly few programmes have much functional integration facilitated through integrated information and communication technologies systems, though all are attempting to implement linked or shared information systems. None of the programmes has fully shared electronic patient records accessible by all professionals involved in care. While Massachusetts General Care Management Program does not have a fully integrated information system, it is perhaps more extensive than most. In particular, many of the programmes have found it difficult to fully integrate data across organizational and professional boundaries with primary care physicians. Most programmes either had partial electronic data sharing capabilities or had ambitions to develop and/or improve such capabilities. Program of Research to Integrate the Services for the Maintenance of Autonomy (Canada) had the most developed fully accessible electronic client chart, although there were some non-affiliated doctors who could not access the information. One of the main obstacles to spreading Program of Research to Integrate the Services for the Maintenance of Autonomy has been implementing the electronic client chart in new localities.

Only Te Whiringa Ora in New Zealand has deployed telemedicine. Monitoring devices are available for use in clients’ homes to measure heart rate, blood pressure, spirometry, pulse oximetry, body temperature, body weight and blood glucose levels. This technology has been primarily used to train patients in self-management, but data are also accessible to clinical staff so they can detect early signs of exacerbations.

#### Funding

All of the programmes started with a developmental or piloting process, often using specially allocated funds (e.g., research grants, growth monies or pilot and demonstration projects). The way in which care is funded reflects different national, regional and local health and social care funding arrangements. In locations where care funding is highly fragmented, such as the USA and Australia, approaches to integrated care have been supported by specific state or federal funding (e.g., a special Medicare demonstration for Massachusetts General Care Management Program, and capital project funding for building a community hub from which HealthOne services were delivered). Massachusetts General Care Management Program has been incorporated into a larger, more ambitious integrated care model known as a Pioneer Accountable Care Organization sponsored by Partners Healthcare.

In less fragmented funding systems, most programmes have created pooled budgets to purchase health and social care collectively, often using a ‘prime contractor’ model in which provider networks are given capitation-based funding to create ‘fully integrated’ purchaser-provider organizations (e.g. Norrtalje, Te Whiringa Ora, Torbay). Exceptions to this approach to pooling funds include Geriant, the specialist provider of dementia services, which has various contracts through which integrated services are provided in different communities, and Program of Research to Integrate the Services for the Maintenance of Autonomy, which has the least integrated and most complex funding model of all.

#### Integrating activities of the programme; participating providers and agencies

##### Approach to care

Integration in these cases was largely the product of improved care co-ordination and management across existing health care providers. Care co-ordination to older people with complex health and social care needs usually involves several core elements including: eligibility criteria for inclusion in care; a single point of referral; a single and holistic care assessment; a care plan; a named care coordinator (or care manager); and support from a multidisciplinary team of care professionals [6]. These elements are almost universally applied across our seven examples, suggesting that these core care coordination features are critical to successful approaches to older people's care *regardless* of the specific organizational, funding or policy context. Of all the care processes used, the most homogenous was the development of single care assessments and subsequent care planning supported by an individual with the power to provide and/or co-ordinate care on behalf of service users.

##### Care providers

An important feature of the seven examples was the differentiation between a ‘core’ group of professionals and/or care teams that provide close, ongoing care to older people and a wider network of care providers who could be drawn upon to support care assessments or improved access to a range of services. Even in Norrtalje, the most fully integrated health and social care approach, a difference existed within the programme between the intensive home care service teams employed in the intervention and the organized network of other health and social care providers. In Massachusetts General Care Management Program, several dedicated teams dealt with different health issues.

The nature of the ‘core group’ differed depending on whether the approach to care focused on care management (direct to service users through multidisciplinary teams) or on care coordination (indirectly across networks of care providers to facilitate access and care coordination). Hence, in Program of Research to Integrate the Services for the Maintenance of Autonomy, HealthOne and Te Whiringa Ora, the ‘core’ team primarily comprised care coordinators working in a close relationship with local community staff or primary care physicians but whose primary role was to support continuity and access to care across a provider network. In the other models of care, the ‘core’ team is multidisciplinary with responsibility for managing and providing a range of care and cure services to older people directly, often within their own home.

##### Care coordinators/care managers

All models have a named care coordinator or care^1^ manager who takes responsibility to support service users (and usually informal caregivers/family members as well). These individuals work to update providers on changes in the patient's status and treatment, and they contact clients to ensure they attend appointments, adhere to their medications, and have access to appropriate services. In many of our examples, care coordinators/managers had face-to-face contact with patients (often in physician offices) and undertook home as well as telephone visits. The frequency and type of contact varied according to the level of need of the individual client. This highly personalized; flexible approach appears to be a common feature of the programmes. Whereas care coordinators tended to be non-clinicians (e.g., health care assistants or social care staff) whose role it is to facilitate access to care services as well as provide a key point of contact; care managers generally had specific training and expertise in caring for older people with complex needs. Hence, care managers not only undertake the care coordination function, but also provide much direct care.

##### Primary care physicians

The literature on care coordination for older people with complex medical problems and/or multimorbidity places high importance on the role of primary care, with many studies suggesting that the more effective approaches have a general practice or primary care physician at the centre of a team-based approach [7–10]. However, in our seven international examples, primary care physicians are rarely part of the ‘core’ team that provide the care coordination or care management function with service users. Experiences across the case sites indicate that it has often been difficult to engage primary care physicians to share data about their patients and to play a proactive role in care delivery, thus becoming a barrier to driving primary- and community-care led integration. Our cases suggest that several factors may contribute to this. Many primary care physicians operate as independent practitioners (indeed, often have both professional and business motives to protect this status) and are not natural partners in collaborative initiatives, even where they might agree with the principle involved. Also many primary care physician practices have intensive workloads, so the time for activities such as care planning or case reviews may be limited. In addition, payment for the work of physicians often sits outside of the wider health and social care system making it problematic to integrate their services more formally with other providers.

#### Evidence of impact, sustainability and spread

It is difficult to provide an overall comparative assessment of the success of our seven programmes because of the variation in the types of evaluations that have been conducted and the data collected and reported. There was no common approach to evaluating or measuring outcomes across cases. Indeed, the degree to which impact measures to evaluate performance and/or care quality were used was highly variable and rarely robust Table 5.

##### Impact

All of the programmes report positive results in terms of improved end user satisfaction and reductions in utilization of hospitals and/or care homes, though some evidence is based pre- and post-utilization, which is subject to regression towards the mean. Of the cases, only Program of Research to Integrate the Services for the Maintenance of Autonomy and Massachusetts General Care Management Program were carefully assessed through funding provided by national research organizations. The Te Whiringa Ora and HealthOne initiatives are smaller and newer than the others and evaluation of their impact is particularly limited. The published evaluations of the other three cases were done retrospectively and lack the rigor of the evaluations conducted in Canada and the USA. Most of the programmes were implemented as service delivery model improvements, and evaluation was a secondary concern.

##### Sustainability and spread

Five of our case examples-Program of Research to Integrate the Services for the Maintenance of Autonomy, Torbay, Massachusetts General Care Management Program, Geriant and Norrtalje - have been in existence long enough to produce some insights into factors that are related to sustainability. Torbay and Norrtalje developed in a context in which health and social care were funded and organized at different levels, and the initiatives required a commitment to change the ‘rules’ in order to allow pooled funding and organizational integration. Although both programmes still exist, subsequent changes to the rules that allowed them to develop mean both programmes face further changes.

Program of Research to Integrate the Services for the Maintenance of Autonomy and MGH began with a specific intervention model that was implemented and evaluated as part of research project funded by a national agency. In these two cases the specific model of care was validated, and the next stage was modification and scaling up to cover larger populations. In these two cases there is evidence of sustainability and generalization. This highlights the importance of defining the intervention, testing and adapting it, and consistently working within the existing system. The Geriant intervention has been able to survive and grow because it has been able to make the move from a small start-up effort to an organization that is able to survive in a commercial and competitive environment. Its sustainability is based on its ability to make an ongoing ‘business case’ for its value.

Most of our examples started life as small-scale demonstration projects or pilots. They have survived, grown and matured over time but this has not been an easy journey. Studies of the cases describe having to work ‘against the grain’ of how care systems or organizations operate often with the need for ‘special measures’ (e.g. legal or financial) to support them. Sustainable models appear to require a stable policy context and positive results, demonstrated through robust evaluation.

##### Transferrable lessons: implications for practice and policy

Our review of seven programmes, like other reviews on the process of integrated care [11], suggests that there is no single ‘best’ approach to integrated care. All of our cases represented a bottom-up initiative rather than top-down structural change. The lack of existing organizational integration had both positive and negative implications. In the absence of existing mechanisms for collaboration, local actors required unified vision, leadership, and hard work to overcome organizational boundaries. Having a unified organization with a common structure has advantages (e.g. single budgets and accountability), but the evidence from our cases suggests that much time and effort is required to merge organizations. In Noortalje, where the greatest efforts for structural integration were made, this activity led to substantial delays in the implementation of practice changes that improved care for the target population.

The case studies of our seven programmes each offered important specific learning points about integrated care. First, it is important to provide stimulus and encouragement for localities without highly prescriptive top-down organizational or clinical rules. Second, start with a clinical/service model rather than structural design and the organizational model. Third, encourage good communication and relationships among those delivering and those receiving care. Finally, remember it takes time to build social capital and foster trust among providers, effectively identify and enrol patients, organize services and begin to see demonstrable changes in outcomes such as readmissions and cost savings.

## Key implications for service providers

Care is ultimately delivered in relationship between patients and providers, and integration is largely the degree to which different providers collaborate with and communicate with each other in caring for the same individual. Key recommendations for provider action include:

- Focus on clinical integration rather than organizational or structural integration.
- Start with a patient-centred model that engages patients and caregivers in the design and implementation of integrated care plans. Provide a single point of entry to enable common assessment.
- Identify a specific care coordinator or care manager. Triage patients according to risk to ensure effective and efficient use of the care manager.
- Establish multidisciplinary teams with well-defined roles in a shared care approach with joint responsibility for care.
- Good communication and trusting relationships among an interdisciplinary collaborative team are essential but also take time to build.

## Key implications for policy/decision-makers

The primary role for policy and decision-makers is to focus on supporting integration activities of the front-line providers. Initiatives to integrate care are bottom-up but ensuring their sustainability and spread requires top-down support. Key areas for policy action include:

- Recognize the importance of addressing this agenda of integrated care for frail older people and make it a system priority.
- Provide stimulus through funding or other means to support the development of local initiatives to improve care for this group of people to enable:
  - Access to wide array of community and social support services delivered by various providers were key features of the interventions in the seven case studies. Pooling previously distinct budgets often enabled this.
  - Care coordinators/care managers, roles that are otherwise largely absent from existing providers.
  - Planning and implementation, particularly where existing fee-for-service systems do not provide payments for these activities. Developing bundled payments may facilitate shared savings but was not fully implemented in any of the cases that we examined.
- Avoid top-down policy that requires structural or organizational mergers.
- Support the implementation of information and communication technologies that can be accessed by multiple different providers. These are a key resource for informational care continuity and to support performance measurement, evaluation and feedback to providers.

## Figures and Tables

**Table 1.**
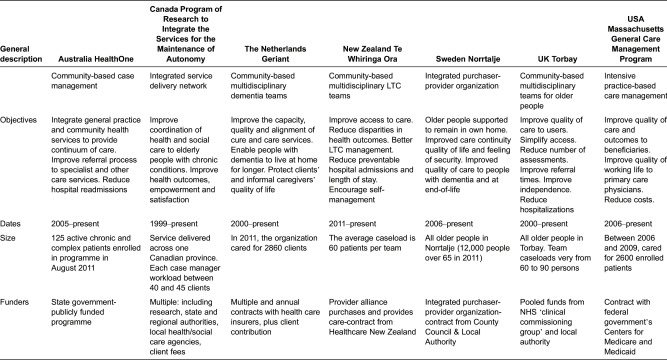
Programme characteristics

**Table 2.**
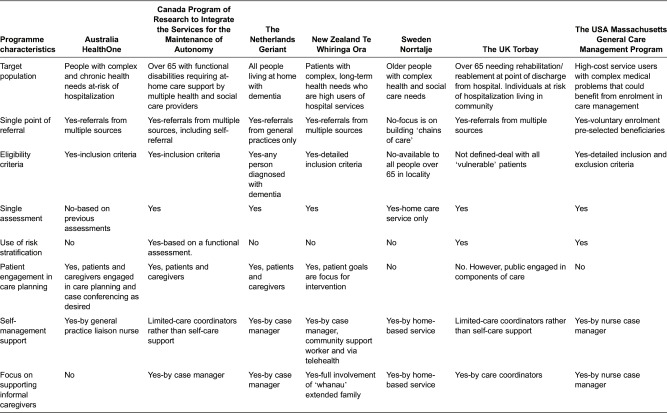
Assessment, care planning and engagement of clients and caregivers

**Table 3.**
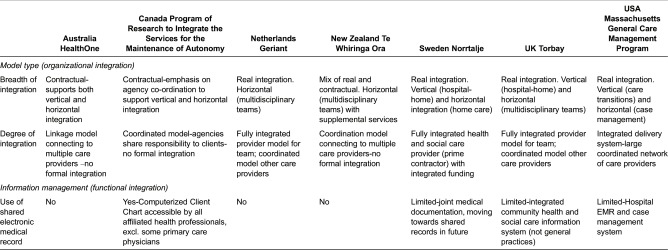
Organizational and functional integration

**Table 4.**
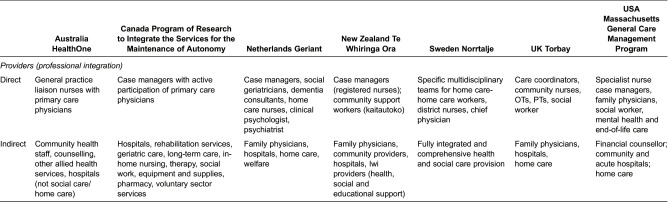
Providers involved directly and indirectly in programmes

**Table 5.**
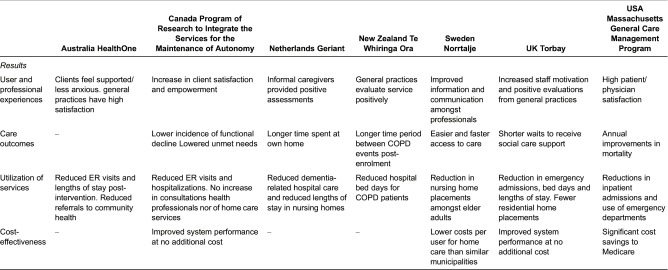
Evidence of outcomes

## Note

1 Some interventions referred to care managers as ‘case’ managers but we adopt the more general care manager title for this role in this report.

## Appendix A

### HealthOne Mount Druitt, Sydney, Australia

HealthOne Mount Druitt provides a hub-and-spoke model of care that operates around a community health centre in a socially disadvantaged area of Western Sydney. The model emphasizes shared care planning to improve coordination of care for older people with complex health needs, reduce unnecessary hospitalizations and ensure appropriate referral to community and specialist health services. Because there is no formal integration of care providers, the model is referred to as ‘virtual’ integrated model. General practice liaison nurses organize multidisciplinary case conferences, coordinate care between various providers involved in the care of the patient, and ensure information is provided about the patient to the general practice or case manager.

### Te Whiringa Ora programme in Eastern Bay of Plenty, New Zealand

Te Whiringa Ora is a collaboration between a community care organization and three newly merged physician practices. The programme began with a focus on chronic respiratory disease and has expanded to include any chronic disease patient with high health care utilization. Patient stories are used to describe the outcomes of the programme. The Te Whiringa Ora programme includes assessment, care coordination, telephone support and telemedicine monitoring as a tool for self-management education. These services are delivered by paired nurse and community-based care coordinators.

### Geriant, Noord-Holland province, the Netherlands

Geriant offers people diagnosed with dementia 24/7 community-based service from teams that include case managers, social geriatricians, psychiatrists, clinical psychologists, dementia consultants and specialized home care nurses. Case managers act as the focal point for clients and their informal caregivers and coordinate services from the team and other network partners including general practices, hospitals, and home care and welfare organizations. Clients have access to a 16-bed short-stay clinic if more intensive treatment or observation is needed.

### Torbay and Southern Devon Health and Care Trust, the United Kingdom

Torbay and Southern Devon Health and Care Trust (known simply as Torbay) was created to commission (purchase) and provide health and social care within a single organization. Care is provided by multidisciplinary health and social care teams with care coordinators that work in geographical ‘zones’ aligned to general practices to provide a range of services that meet the needs of older people after discharge from hospital. More recently pro-active case management of at-risk older people using predictive risk tools has provided an added capability to intervene before hospitalizations occur. These teams also provide ongoing care and support in the home environment.

### The Norrtalje Model, Sweden

The Stockholm County Council and the Norrtalje Local Authority, which are responsible, respectively, for health care and social care services for the Norrtalje population, formed joint governing committee to be responsible for both types of services. This committee owns and directs a public company that is responsible for purchasing and delivering care. The model is characterized by (1) funding responsibilities for the whole population within the county, (2) increased focus on health promotion for the population and (3) a common and integrated health and social care organization to achieve greater patient and user benefit. There was an emphasis on using care managers and on developing pathways and plans for transitions in and out of hospital from nursing homes to hospital.

### The Massachusetts General Care Management Program, Boston, USA

The Massachusetts General Care Management Program started as a demonstration at one academic site and has evolved-first by expanding to more sites (including non-academic settings) and then as a component of a new Pioneer Accountable Care Organization. The Massachusetts General Care Management Program is focused on high-cost patients with multiple hospitalizations and multiple chronic conditions who are offered care that is integrated by a case manager embedded in a primary care practice. Practice-based case managers have intensive, one-on-one relationships with their patients through in-person interactions at the physician's office or when hospitalized, periodic telephone calls (at least once every 4–6 months) and home visits as needed.

### The Program of Research to Integrate the Services for the Maintenance of Autonomy Quebec, Canada

Program of Research to Integrate the Services for the Maintenance of Autonomy began in Quebec in 1999. Its objective was to implement an integrated service delivery network to improve the health, empowerment and satisfaction of frail older people and to change health and social service utilization without increasing caregiver burden. Its key components are service coordination, single entry point, case management, a single functional assessment tool, individualized service plans and a shared information system. Since 2001, the Quebec Ministry of Health and Social Services made implementing the six features of the Program of Research to Integrate the Services for the Maintenance of Autonomy approach a province-wide goal in the programme now known as RSIPA (French acronym).
